# Maternal nutritional imbalance during pregnancy and the development of fetal-origin cardiovascular diseases

**DOI:** 10.3389/fnut.2026.1717069

**Published:** 2026-01-21

**Authors:** Shihua Cui, Fengying Deng, Muxue Lu, Meihua Zhang, Zedong Yang, Yuchen Ma, Linlin Fan, Qinqin Gao, Dairong Feng

**Affiliations:** 1 School of Life Science and Technology, Shandong Second Medical University, Weifang, China; 2 Institute for Fetology, The First Affiliated Hospital of Soochow University, Suzhou, China; 3 Key Laboratory of Maternal and Fetal Medicine of National Health Commission of China, Shandong Provincial Maternal and Child Health Care Hospital Affiliated to Qingdao University, Jinan, China

**Keywords:** adverse dietary patterns, cardiovascular disease, offspring, overnutrition, undernutrition

## Abstract

The prenatal period is a critical window for cardiovascular development in offspring. Accumulating evidence demonstrates that maternal nutritional imbalances during pregnancy—encompassing undernutrition, overnutrition, and specific nutrient deficiencies—elicit adverse adaptations in fetal vascular systems, thereby predisposing offspring to cardiovascular disease (CVD) in later life. This review synthesizes current knowledge on the impact of macronutrient (e.g., high-sugar, high-fat diets) and micronutrient (e.g., vitamin D, folate) imbalances, as well as exposures to alcohol and caffeine, on offspring cardiovascular health. Key mechanisms such as epigenetic regulation (e.g., DNA methylation, histone modifications), oxidative stress, and endothelial dysfunction are discussed. Furthermore, we highlight future research directions and potential early nutritional interventions aimed at mitigating long-term cardiovascular risks and breaking the cycle of intergenerational metabolic disease. By integrating mechanistic insights and epidemiological evidence, this review underscores the importance of optimizing maternal nutrition as a pivotal public health strategy for preventing offspring CVD.

## 1 Introduction

Cardiovascular disease (CVD) remains the leading cause of global mortality and disability, accounting for over 18 million annual deaths—32% of all non-communicable disease-related mortality (1). Traditional preventive approaches have focused predominantly on adult risk factors such as smoking, obesity, and hypertension. However, growing epidemiological evidence suggests that a substantial proportion of CVD cases originate from adverse environmental exposures during fetal development (2–4). This concept was first systematically articulated by Barker as the “fetal origins hypothesis” and later expanded into the Developmental Origins of Health and Disease (DOHaD) framework. The DOHaD posits that suboptimal intrauterine conditions can induce developmental reprogramming, leading to persistent structural and functional changes in fetal organs and an increased lifelong susceptibility to CVD (5–7). As research in this area advances, the fetal origins of CVD have emerged as an important interdisciplinary field. Investigating the underlying mechanisms not only deepens our understanding of CVD etiology but also opens a critical window for early-life interventions.

Maternal nutrition during pregnancy plays a fundamental role in fetal programming and long-term cardiovascular health (8–10). Beyond supplying essential nutrients—proteins, lipids, vitamins, and minerals—maternal diet conveys nutrient-derived signals across the placenta, influencing gene expression, cellular differentiation, and organogenesis, with lasting consequences for offspring metabolism and vascular function (11, 12). Modern dietary patterns exacerbate these effects. Overnutrition, driven by rising rates of pre-pregnancy obesity and excessive gestational weight gain, contributes to gestational diabetes and fetal metabolic dysfunction through hyperglycemia, dyslipidemia, and inflammation (13, 14). Concurrently, micronutrient deficiencies (e.g., iron, folate, vitamin D) remain widespread due to dietary inadequacy or socioeconomic barriers, impairing placental function and epigenetic regulation, and elevating CVD risk in offspring (11, 15, 16). In addition, emerging dietary behaviors introduce additional risks. High caffeine intake, prevalent in fast-paced societies, has been epidemiologically associated with adverse birth outcomes. However, the specific mechanisms through which caffeine influences the fetal cardiovascular system remain poorly understood (17, 18). Similarly, excessive salt consumption—common in processed and restaurant foods—has been linked in animal and human studies to programming effects on blood pressure and renal development (19, 20). Alcohol use during pregnancy, even at low levels, continues to pose significant teratogenic risks to cardiac structure and vascular function (21).

Encouragingly, increasing public and scientific awareness has spurred rigorous epidemiological and mechanistic research, particularly in the fields of epigenetics and metabolic programming, thereby strengthening the evidence base linking maternal nutrition to cardiovascular outcomes in offspring (8). Key markers such as blood pressure, vascular reactivity, lipid metabolism, and insulin sensitivity now show consistent associations with prenatal nutritional exposures (4, 22, 23). This review synthesizes current evidence on the role of maternal nutrition—encompassing undernutrition, overnutrition, and specific dietary components—in the developmental origins of CVD. By integrating mechanistic insights and clinical implications, we aim to inform effective preventive strategies, guide public health practice, and ultimately improve cardiovascular health across generations.

## 2 Literature search strategy

A systematic literature search was performed across three electronic databases: PubMed, Web of Science Core Collection, and Embase, covering records from their inception to November 2025, with no language restrictions applied initially. The search strategy was developed in consultation with a subject librarian, combining Medical Subject Headings (MeSH) terms and free-text keywords centered on three core concepts: maternal exposures (e.g., hyperglycemia, high-fat diet), fetal or developmental programming, and cardiovascular outcomes in offspring. Additional studies were identified by manually screening the reference lists of relevant articles. Study selection followed the Preferred Reporting Items for Systematic Reviews and Meta-Analyses (PRISMA) guidelines. Eligible studies were original research articles investigating the association between specific maternal exposures and offspring cardiovascular outcomes while exploring underlying developmental programming mechanisms. Data were extracted using a standardized form and synthesized narratively, with evidence organized into two main sections: epidemiological findings from human studies and mechanistic insights from experimental research.

## 3 Excessive maternal nutrition during pregnancy

Excessive maternal nutrition during pregnancy commonly stems from a chronic high-calorie diet, characterized by abundant refined sugars (e.g., sucrose and fructose) and foods high in saturated or trans fats—such as fried foods and processed meats. These dietary patterns induce metabolic disturbances, including dysregulated glucose homeostasis, abnormal lipid profiles, and chronic inflammation. Such alterations disrupt fetal cardiovascular development via placenta-mediated metabolic reprogramming and epigenetic modifications (10, 24). Maternal body weight serves as a direct reflection of nutrient intake. A large Swedish cohort study demonstrated a dose-dependent association between maternal obesity and offspring CVD risk. Specifically, children of severely obese mothers exhibited a 60% increased CVD risk compared to those of normal-weight mothers (25). Studies on prenatal high-glucose exposure associate elevated maternal carbohydrate intake with higher offspring blood pressure, including significant increases in both systolic and diastolic pressures (26, 27). Similarly, a prospective cohort study in the United States suggested that the intake of a high-fat, high-sugar, and high-sodium diet in early pregnancy would significantly increase the compound risk of adverse pregnancy outcomes, including preeclampsia, gestational diabetes, preterm delivery, and small for gestational age (SGA) infants (28). Additionally, it should be noted that combined exposure to high sugar and high fat—as typified by Western-style diets—exerts additive adverse effects on offspring cardiovascular health, directly induces maternal metabolic dysfunction, alters maternal-fetal nutrient partitioning, and imposes long-term consequences on maternal and infant health (29).

Prenatal high glucose exposure contributes to fetal-origin CVD through multiple pathological mechanisms, such as metabolic dysregulation, epigenetic alterations, oxidative stress, and inflammation. Evidence from animal studies supports these pathways. For instance, a hyperglycemic intrauterine environment disrupts normal cardiac development by inducing fetal cardiac hypertrophy—particularly in late gestation—and impairing key processes including cardiac neural crest cell migration, conotruncal formation, and endocardial cushion mesenchymal development (30). Moreover, high glucose alters the expression of specific miRNAs (e.g., miR-181a) in fetal endothelial cells, resulting in offspring endothelial dysfunction (31). In rat models, maternal diabetes influences fetal cardiac angiogenesis and oxidative stress via miRNA-mediated gene silencing (32). Elevated levels of TNF-α and IL-6 have also been detected in placental tissues under hyperglycemic conditions, correlating with increased placental weight, reduced fetal weight, and diminished placental efficiency (33). Both human and animal studies further indicate that offspring of mothers with gestational diabetes display impaired vasculogenic capacity in endothelial progenitor cells, significantly increasing their susceptibility to CVD in adulthood (32).

Similarly, the pathological mechanisms by which prenatal high-fat exposure contributes to fetal-origin CVD also involve multiple pathways, including dysregulated lipid metabolism, altered placental function, oxidative stress, and epigenetic regulation. These are discussed below with support from relevant animal studies (34). Prenatal high-fat exposure leads to abnormally elevated lipid levels in the fetal circulation. Since the fetal heart primarily relies on carbohydrates for energy, premature exposure to high lipid levels alters myocardial metabolic preference and suppresses cardiac energy metabolism (35, 36). A study indicates that although lipid uptake-related genes (such as CD36 and Carnitine Palmitoyltransferase 1A/B) are upregulated in fetal cardiomyocytes following lipid exposure, overall metabolic activity is reduced, ultimately impairing cardiac function (37). Maternal high-fat diets also alter the expression of placental lipid transporters (e.g., ATP-Binding Cassette Transporters), influencing fetal lipid exposure (38). In rat models, maternal lipopolysaccharide administration disrupts the expression of these transporters, resulting in adult offspring that are more susceptible to diet-induced CVD (39, 40). Furthermore, a high-fat diet induces oxidative stress and activates inflammatory pathways (such as NF-κB) in both the mother and fetus, leading to cardiomyocyte apoptosis and endothelial dysfunction (35). Offspring of mice fed a high-fat diet during gestation exhibit elevated blood pressure and cardiac hypertrophy in adulthood, even when maintained on a normal diet (34). This effect may be linked to programmed alterations in the leptin signaling pathway. Maternal high-fat exposure appears to induce epigenetic modifications—such as DNA hypomethylation—that affect the expression of metabolic genes in the fetal heart, increasing the offspring’s susceptibility to hypertension and atherosclerosis in later life (34). Additionally, following maternal exposure to a high-fat diet, offspring exhibit sex-specific cardiovascular responses. For instance, one study suggests that male offspring demonstrate more severe alterations in cardiovascular function (such as elevated blood pressure and enhanced vasoconstriction), while female offspring may adapt or compensate through different mechanisms (41).

The Western-style diet is an obesogenic dietary pattern characterized by high fat and sugar content and elevated energy density. Beyond macronutrient imbalance, a Western-style diet triggers a cascade of adverse metabolic and physiological effects. Notably, exposure during the critical period of pregnancy can impose profound detrimental consequences on the health of both the mother and offspring (29). The pathogenic mechanisms underlying combined sugar and fat exposure are notably complex (Table 1).

**Table 1:** Pathogenic mechanisms of maternal western-style diet (high fat/high sugar) exposure on offspring cardiovascular development.

Study model	Key findings	Specific pathological manifestations/effects	Reference
Rat model	Maternal diabetes combined with a high-sugar/high-fat diet directly impairs fetal cardiac mitochondrial function and metabolic homeostasis.	Impaired fetal cardiac mitochondrial respiration; increased oxidative stress (e.g., elevated MDA); lipid accumulation in cardiomyocytes.	(35)
JEG-3 cell line	Combined high-glucose and high-fat exposure directly induces oxidative stress and metabolic disturbance in placental cells, suggesting a placenta-mediated impact on the fetus.	Dysfunctional mitochondria in placental trophoblasts; decreased antioxidant enzyme (SOD) activity; increased release of cell damage marker (LDH).	(42)
Rat model	High-fat diet exacerbates the abnormal metabolic programming in fetal myocardium induced by maternal diabetes.	Abnormal expression of key metabolic enzymes (e.g., CPT-1, PDK4) in fetal cardiomyocytes; Impaired mitochondrial fatty acid oxidation capacity.	(43)
Rat model	Maternal diabetes combined with a high-fat diet triggers placental inflammation, subsequently damaging the structure and function of fetal major blood vessels.	Increased release of placental inflammatory factors (e.g., TNF-α, IL-6) leads to fetal aortic endothelial dysfunction and early atherosclerotic-like changes.	(44)
Rat model	Combined sugar and fat exposure exerts particularly pronounced detrimental effects on offspring vascular reactivity.	Significantly impaired vascular constriction in offspring.	(45)
Rat model	Maternal high-fat diet disrupts normal placental nutrient delivery, metabolism, and barrier function, ultimately reducing nutrient supply to the fetus and impairing its development.	Dysregulated placental lipid transport (e.g., increased VLDL secretion); dysfunction of glucose transporters; placental lipid accumulation and oxidative stress.	(43, 46)

In summary, maternal high-sugar and high-fat diets disrupt normal cardiovascular development and functional programming in the fetus. Excessive macronutrient intake is particularly characterized by inducing profound metabolic disturbances (e.g., hyperglycemia, dyslipidemia) and mitochondrial dysfunction in the fetal heart, which, coupled with diet-specific epigenetic reprogramming, sets the stage for offspring CVD risk. These findings underscore the importance of nutritional intervention and metabolic management during pregnancy as critical strategies for preventing fetal-origin CVDs.

## 4 Inadequate nutrition during pregnancy

Inadequate nutrition during pregnancy is a key driver of fetal-origin cardiovascular disease, primarily involving deficiencies in macronutrients (such as carbohydrates, lipids, and proteins) and micronutrients (including folate, vitamin B12, zinc, copper, and selenium) (47).

### 4.1 Inadequate macronutrient intake during pregnancy

Inadequate macronutrient intake during pregnancy has been identified as a significant risk factor for fetal-origin CVD. Fetal growth restriction (FGR), a common consequence of such deficiency, is strongly associated with an increased risk of cardiovascular disorders in adulthood. Epidemiological data indicate that adults who experienced FGR have a 2–3 times higher incidence of hypertension, atherosclerosis, and chronic kidney disease compared to those with normal birth weight (48, 49). Furthermore, both animal studies and human observational research suggest that insufficient maternal caloric or protein intake during gestation can lead to a “thrifty phenotype” adaptation in the fetus. This metabolic reprogramming prioritizes brain development at the expense of cardiovascular health, resulting in long-term consequences such as insulin resistance, endothelial dysfunction, and elevated blood pressure (49, 50).

Among the forms of macronutrient deficiency, maternal protein deficiency (MPD) and maternal caloric restriction (MCR) are the most extensively studied in relation to developmental cardiovascular programming. The underlying mechanisms through which MPD and MCR contribute to fetal-origin CVD involve multi-level physiological and molecular alterations, which can be summarized as follows:

MPD during critical developmental windows disrupts cardiovascular structure and physiological function. Specifically, MPD leads to aberrant protein expression in cardiac tissue, impairing myocardial growth and energy metabolism, thereby increasing the risk of CVD in adulthood (51). For instance, animal studies have shown that MPD in pregnant rats induces FGR, alters the cardiac proteome, and results in structural and functional cardiac abnormalities (52). MPD is also associated with mitochondrial dysfunction and increased oxidative stress, contributing to cardiac tissue damage (53). In both cerebral and cardiac tissues, protein deficiency disrupts mitochondrial metabolic balance, promotes the accumulation of free radicals, and induces oxidative damage. These changes impair energy production and cellular function in cardiomyocytes, leading to compromised cardiac bioenergetics and ultimately manifesting as cardiomyocyte hypertrophy and cardiac dysfunction (51, 54). Additionally, protein deficiency causes permanent alterations in renal function, including reduced sodium excretion and dysregulation of the renin–angiotensin–aldosterone system (RAAS) (55). These alterations result in diminished sodium handling capacity in offspring, promoting water and sodium retention and facilitating the development of hypertension (56, 57). Epigenetic mechanisms may also play a role. Evaluations of adult male offspring exposed to prenatal protein deficiency show altered cardiac morphology—including abnormal left ventricular microRNA expression and dysregulation of related target genes—supporting the involvement of epigenetic regulation in cardiac hypertrophy and dysfunction (58). Moreover, studies have indicated that there are sex differences in the cardiovascular disease risk among offspring induced by MPD. Specifically, the incidence of cardiovascular diseases in female offspring is significantly lower than that in male offspring. This phenomenon is primarily attributed to the protective effect of estrogen (53).

MCR significantly impairs placental development and function. Studies indicate that a 50% reduction in caloric intake disrupts placental angiogenesis by downregulating miRNA-regulated pathways—such as those involving Vegfa and Tgfβ—leading to impaired vascularization, increased oxidative stress, and elevated apoptosis (59). MCR also compromises trophoblast function, reducing the transport of critical nutrients such as glucose across the placenta, which directly contributes to fetal hypoglycemia and nutrient deficiency (48). These placental alterations are accompanied by profound structural and metabolic reprogramming in the fetal heart. Studies in sheep models demonstrate that severe FGR resulting from MCR is associated with ventricular wall thickening, reduced heart weight, and disordered myocardial fiber arrangement—structural abnormalities linked to an increased risk of CVD in adulthood (60, 61). In rats, maternal nutrient restriction leads to reduced protein synthesis and downregulated mitochondrial protein expression in the neonatal heart (52). Furthermore, MCR induces fetal cardiac mitochondrial dysfunction, characterized by decreased complex I activity, impaired ATP production (50), and reduced fatty acid oxidation. This metabolic shift forces greater reliance on glucose for energy, exacerbating energetic deficiency—a pattern that persists into adulthood, as observed in sheep FGR models (61). MCR also promotes oxidative stress and cellular damage. In guinea pig models, maternal hypoxia—a common consequence of caloric restriction—results in failed placental vascular remodeling, causing chronic fetal hypoxia and subsequent cardiomyocyte apoptosis and cardiac fibrosis (62). The MCR-exposed placenta exhibits reduced antioxidant capacity, leading to increased reactive oxygen species (ROS) that enter fetal circulation and directly damage the developing cardiovascular system. Notably, vitamin C supplementation in animal models has been shown to partially mitigate this damage (56). Endocrine and neuroregulatory dysfunctions also contribute to MCR-related cardiovascular programming. MCR induces structural changes in the fetal hypothalamus, leading to sustained overexpression of glucocorticoid receptors and adult-onset blood pressure dysregulation (63). Offspring exposed to MCR exhibit reduced baroreflex sensitivity and sympathetic overactivation, which promote ventricular remodeling (64). Finally, MCR induces persistent epigenetic changes. Rat studies demonstrate that maternal caloric restriction leads to lasting alterations in the expression of cardiovascular-related genes through DNA methylation. These include upregulation of RAAS components and suppression of insulin signaling pathways (64, 65), resulting in a heightened predisposition to hypertension and insulin resistance in adulthood.

Collectively, macronutrient deficiency (MPD and MCR) programs offspring CVD primarily through structural compromises in the heart and kidneys, severe placental insufficiency, and associated metabolic and neuroendocrine reprogramming, distinct from the mechanisms highlighted in overnutrition.

### 4.2 Micronutrient deficiency during pregnancy

Epidemiological studies, primarily based on prospective cohort and case–control studies, indicate that deficiencies in specific micronutrients during pregnancy are associated with an increased risk of long-term CVD in offspring. For instance, low maternal folate levels have been linked to elevated blood pressure, impaired endothelial function, and increased carotid intima-media thickness in children and adolescents (66). Vitamin D deficiency during pregnancy has been negatively correlated with early cardiovascular risk markers in offspring, including hypertension, left ventricular hypertrophy, and arterial stiffness (67). Evidence regarding other micronutrients such as iron, zinc, and vitamin B12 remains limited, though some studies suggest that deficiencies may contribute to elevated blood pressure or metabolic abnormalities in offspring (68).

The mechanisms through which maternal micronutrient deficiency contributes to fetal-origin CVD can be summarized as follows, based on current evidence (Table 2).

**Table 2:** Mechanisms of maternal micronutrient deficiencies to fetal-origin cardiovascular disease.

Mechanistic category	Core action/target	Consequent pathophysiological effects	Reference
Epigenetic dysregulation	One-carbon metabolism (dependent on folate and vitamin B12 as critical cofactors), which regulates processes such as DNA methylation.	Alters fetal gene expression patterns, disrupting the developmental programming of cardiovascular systems and leading to endothelial dysfunction and reduced cardiac adaptive capacity in adulthood.	(69–72)
Oxidative stress and inflammatory imbalance	Core components of antioxidant enzymes (e.g., zinc and selenium in superoxide dismutase and glutathione peroxidase).	Elevates fetal oxidative stress, impairs endothelial function, and activates inflammatory pathways (e.g., NF-κB), resulting in a chronic low-grade inflammatory state and permanent alterations in fetal vascular tone regulation.	(16, 73)
	Mitochondrial function (copper-dependent enzymes such as cytochrome c oxidase).	Copper deficiency exacerbates mitochondrial dysfunction and reactive oxygen species accumulation, compromising cardiomyocyte energy metabolism.	(16, 74)
Impaired vascular development and function	Angiogenesis and vascular remodeling (involving iron and folate).	Iron deficiency induces maternal hypoxia, stimulating excessive fetal erythropoietin production, which promotes abnormal vascular proliferation, collagen deposition in vessel walls, and increased arterial stiffness in adulthood.	(75, 76)
	Vascular wall structure (zinc influences collagen-to-elastin ratio).	Prenatal zinc deficiency increases the aortic collagen-to-elastin ratio and reduces vascular compliance, with these structural alterations persisting postnatally.	(16)
Altered cardiac morphology and metabolic programming	Myocardial structure (copper is essential for lysyl oxidase activity, affecting extracellular matrix cross-linking).	Copper deficiency impairs myocardial extracellular matrix cross-linking, leading to ventricular wall thinning and systolic dysfunction.	(77, 78)
	Cardiac growth (zinc regulates insulin-like growth factor signaling).	Zinc deficiency disrupts cardiomyocyte proliferation, resulting in reduced heart size—a structural abnormality associated with increased risk of heart failure in later life.	(16)
	Metabolic programming (influenced by multiple micronutrients).	May dysregulate fetal hepatic cholesterol metabolism, thereby promoting early atherogenic pathways.	(76)

In summary, maternal micronutrient deficiency contributes to fetal-origin CVD. A key distinctive aspect of micronutrient deficiencies lies in their disruption of one-carbon metabolism (e.g., folate, B12) and their role as cofactors for antioxidant enzymes (e.g., Zn, Se, Cu), leading to unique patterns of epigenetic dysregulation and oxidative damage that impair cardiovascular development. These findings highlight potential molecular targets for nutritional interventions during pregnancy, such as combined supplementation with zinc, selenium, and antioxidants, which may more effectively disrupt these pathological pathways.

## 5 Specific dietary behaviors during pregnancy

Specific dietary behaviors during pregnancy represent a significant global public health concern. Among these, alcohol abuse, high salt intake, and excessive caffeine consumption are particularly notable due to their widespread prevalence and established developmental toxicity. These factors are considered modifiable risk factors for fetal-origin CVD (79–81). Epidemiological data indicated that despite increasing public health warnings, approximately 10% of pregnant women worldwide report alcohol consumption during pregnancy, with rates reaching as high as 25% in some regions. Exposure is especially common during the early stages of pregnancy, often before pregnancy is recognized (79). High salt (sodium) intake is even more prevalent, largely driven by the widespread consumption of processed foods and cultural dietary patterns. In the majority of regions, daily sodium intake among pregnant women significantly exceeds the WHO-recommended limit of <5 g of salt per day, with particularly high levels observed in East Asia and Western countries where high-salt diets are common (81). Similarly, high caffeine consumption (>200 mg per day) affects a substantial proportion of pregnant women, with an estimated 15–30% reporting regular intake from sources such as coffee, tea, or energy drinks (82, 83). Against this backdrop, the following sections will focus on examining the specific effects of prenatal alcohol exposure, high-salt diets, and caffeine exposure on the cardiovascular health of offspring.

### 5.1 Prenatal alcohol exposure

**Figure 1:**
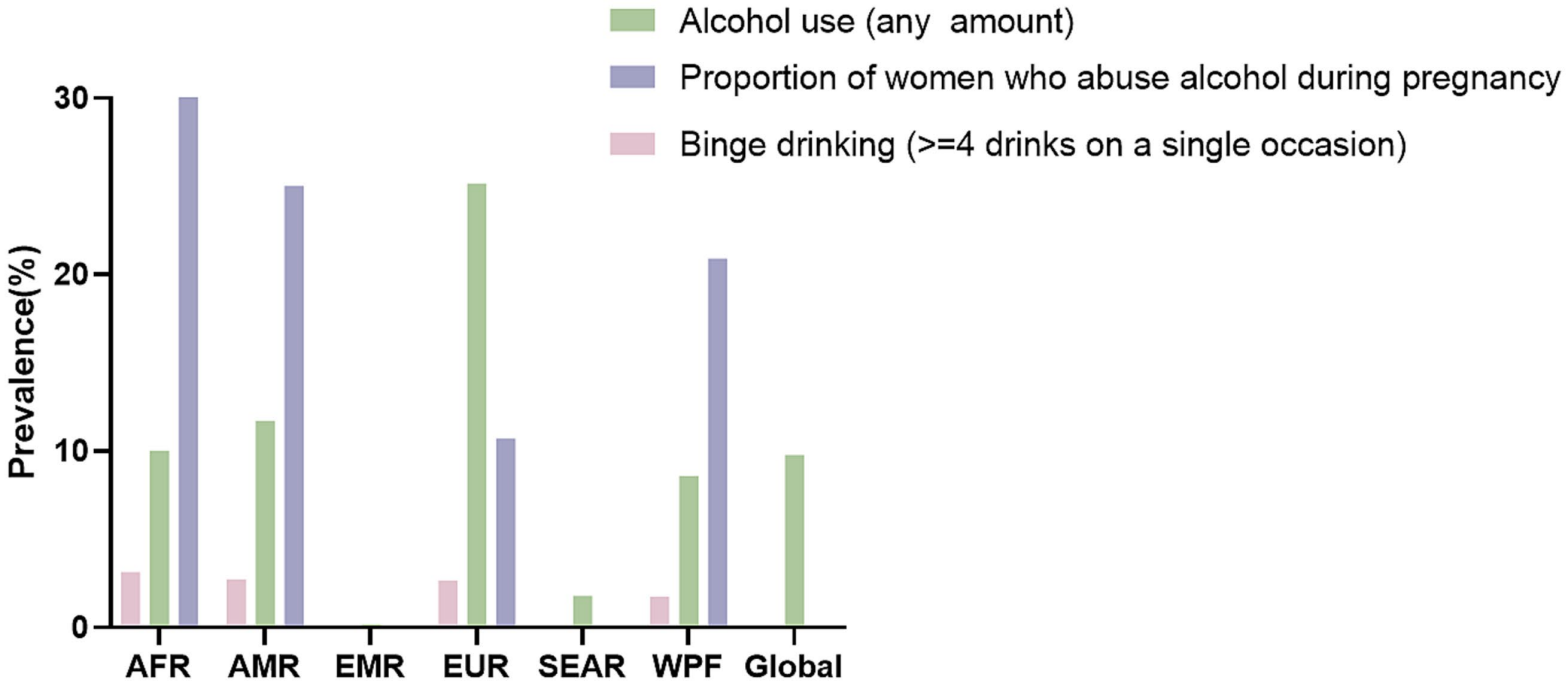
The prevalence (%) of any amount of alcohol use and binge drinking during pregnancy and the estimated proportion of women who binge drank during pregnancy by World Health Organization region. AFR = African Region, AMR = Region of the Americas, EMR = Eastern-Mediterranean Region, EUR = European Region, SEAR = South-East Asia Region, WPR = Western Pacific Region (The prevalence of any amount of alcohol use during pregnancy is inclusive of the prevalence of binge drinking during pregnancy). Created using Figdraw.com.

Prenatal alcohol exposure (PAE), referring to fetal contact with ethanol through maternal consumption, represents a significant global public health issue (84). According to the WHO reports, a considerable proportion of women continue to consume alcohol during pregnancy, including episodes of heavy drinking (85) (Figure 1). PAE is characterized by several parameters, including alcohol dosage, exposure pattern (e.g., binge versus chronic drinking), timing during gestation (e.g., first or third trimester), and duration (86). It is important to note that no safe threshold of alcohol consumption during pregnancy has been established, and any exposure may pose risks to the fetus (79). While a majority of the current evidence derives from animal studies, further large-scale prospective cohort studies in human populations are needed.

Regarding pathogenic mechanisms, rat models demonstrate that administration of ethanol (4.5 g/kg body weight) during prenatal and early postnatal stages leads to increased markers of oxidative stress (e.g., malondialdehyde) and elevated pro-inflammatory cytokines (e.g., TNF-α) in cardiac tissue, accompanied by enhanced cardiomyocyte apoptosis (87). Offspring exposed to PAE exhibit aortic medial thickening, disrupted elastic fibers, increased collagen deposition, and impaired endothelium-dependent vasodilation at 21 and 90 days after birth, suggesting an elevated risk for early atherosclerotic lesions. These findings indicate that ethanol induces myocardial damage through oxidative stress, inflammatory responses, and apoptosis (88, 89). Additionally, mouse models reveal that PAE disrupts the transcription of genes involved in calcium signaling and cardiac metabolism, resulting in abnormal myocardial structure and function (90). Another rat study suggests that alcohol exposure leads to fetal anemia by upregulating hepcidin and erythropoietin, thereby disrupting iron homeostasis. This functional iron deficiency may contribute to subsequent cardiovascular dysfunction, such as reduced cardiac output related to anemia (91, 92).

Ethanol exposure has also been shown to directly induce structural remodeling (e.g., wall thickening) and functional impairment (e.g., endothelial dysfunction) in the fetal aorta, associated with pro-inflammatory factors and oxidative stress (93). Specifically, aortas from PAE offspring are more susceptible to atherosclerosis, with inflammatory mediators promoting cell adhesion and endothelial injury (89). In *ex vivo* models, such as pressurized cerebral arteries, ethanol-induced vasodilation was mediated through the activation of cannabinoid receptors (CB1 and CB2), as confirmed by inhibitor experiments (94). Similar mechanisms affecting vascular reactivity have been observed in baboon models, providing insight into potential mechanisms underlying human cerebrovascular pathology (84). These findings collectively indicate that PAE promotes vascular disease through inflammatory and remodeling processes. In the rat studies mentioned, aortic abnormalities were partly attributed to cytokine-mediated vascular remodeling (89). Epigenetic mechanisms have also been implicated in mouse models, where PAE altered DNA methylation patterns and affected cardiac signaling pathways (95).

In summary, PAE induces cardiovascular abnormalities in animal models. Beyond common pathways, alcohol exhibits direct teratogenic effects, causing specific structural defects in the heart and aorta, and uniquely disrupts calcium signaling and iron homeostasis, contributing to its cardiotoxic profile.

### 5.2 High salt intake during pregnancy

Prenatal high salt intake (PHSI) is commonly defined as a daily consumption exceeding 5.75 g (based on 24-h urinary sodium excretion), with very high intake classified as greater than 10.25 g per day (96). Numerous studies highlight that salt intake among pregnant women exceeds the WHO-recommended limit of 5 g/day in many populations worldwide (97, 98). A prospective cohort study involving 184 pregnant women used 24-h urine collection to assess sodium excretion and identified high salt intake as a significant predictor of pregnancy-induced hypertension (81), suggesting a potential indirect increase in offspring cardiovascular risk. Another cohort study found that salt intake exceeding 6 g/day significantly elevated the risk of preeclampsia (20), a condition linked to placental dysfunction and adverse perinatal outcomes that may contribute to fetal programming of CVD (19).

The mechanisms through which PHSI contributes to fetal-origin CVD involve several pathways. PHSI suppresses local RAAS activity in the fetoplacental unit and reduces the levels of placental vascular endothelial growth factor (VEGF), leading to impaired placental vascular function (96). An animal study has shown that offspring of dams fed a high-salt diet exhibit coronary arterial wall thickening, endothelial degeneration, and altered expression of key RAAS components (such as AT1R downregulation and ACE2 upregulation), resulting in enhanced vascular sensitivity to angiotensin II (99). These offspring also demonstrate structural renal abnormalities (e.g., enlarged glomerular area) and myocardial fibrosis soon after birth, accompanied by local RAAS overactivation, indicating early impairment of organ development (100). Additionally, adult male offspring from high-salt-fed dams exhibit impaired endothelium-dependent vasodilation in response to acetylcholine in mesenteric arteries, associated with increased superoxide production and reduced nitric oxide bioavailability (101). Elevated levels of ROS and decreased antioxidant enzyme activity have also been observed in vascular tissues, contributing to oxidative damage (102). Another rat model revealed that prenatal high salt intake leads to elevated resting blood pressure and increased salt sensitivity in adulthood. This effect is mediated by inflammatory activation (e.g., increased TNF-α and IL-1β) in the hypothalamic paraventricular nucleus (PVN) and sympathetic overactivity, promoting the development of hypertension in offspring (103).

In summary, PHSI interferes with fetal cardiovascular developmental programming. A hallmark of high salt exposure is its pronounced suppression of the local placental RAAS and its programming of sustained salt sensitivity in offspring, often mediated by central inflammatory activation and sympathetic overactivity, leading to hypertension.

### 5.3 High caffeine intake during pregnancy

Current epidemiological studies commonly define high caffeine intake during pregnancy as consumption exceeding 200 mg per day. Multiple reports indicate that prenatal high-caffeine intake (PHCI) is associated with an increased risk of SGA and LBW infants (80). Both SGA and LBW are established risk factors for cardiovascular disease in later life, likely mediated through impaired development of key organs such as the heart and blood vessels. Data from the Finnish KuBiCo cohort showed that caffeine intake of 51–200 mg/day during early pregnancy was associated with an 87% increased risk of SGA, while intake exceeding 200 mg/day increased the risk by 51% (104). A Japanese study also found that caffeine consumption above 200 mg/day during the second trimester significantly elevated the risk of delivering a LBW infant (105).

Animal studies provide mechanistic insights into these observations. Rat models demonstrate that PHCI leads to elevated total cholesterol levels in adult offspring, increasing the risk of hypercholesterolemia. This metabolic reprogramming appears to be driven by caffeine-induced epigenetic modifications—such as changes in DNA methylation of key regulatory genes, including SREBP2, which is involved in cholesterol metabolism (106, 107). Another study reported cerebrovascular dysfunction in 24-month-old offspring following PHCI, resulting from caffeine-mediated inhibition of placental 11β-HSD2 enzyme activity. This leads to fetal overexposure to maternal glucocorticoids, activating downstream metabolic pathways that contribute to dysregulated cholesterol metabolism and cardiovascular dysfunction in adulthood, suggesting an elevated long-term risk of cerebrovascular disease (108). Mouse models further reveal that PHCI (equivalent to 2–4 cups of coffee per day in humans) induces cardiac functional abnormalities in offspring, with evidence suggesting these effects may be transmitted across generations, highlighting the persistent impact of caffeine on the cardiovascular system (109). Another experimental study showed that PHCI (120 mg/kg/day) significantly reduces placental maternal blood perfusion, triggering compensatory fetal vascular proliferation and disrupting angiogenic balance. This placental hypoperfusion and hypoxia impair fetal cardiovascular development via hypoxia-inducing signaling pathways (e.g., HIF), ultimately leading to fetal growth restriction and establishing a foundation for cardiovascular disease in later life (110, 111).

Collectively, these findings indicate that prenatal caffeine exposure contributes to fetal-origin cardiovascular disease. A predominant mechanism specific to caffeine is the inhibition of placental 11β-HSD2, resulting in fetal overexposure to maternal glucocorticoids, and the induction of placental hypoperfusion, which jointly drive adverse metabolic and cardiovascular outcomes.

These factors have been identified as significant modifiable risk factors for fetal-origin cardiovascular disease, with far-reaching and complex implications. Through mechanisms such as oxidative stress, inflammatory activation, epigenetic reprogramming, and placental dysfunction, suboptimal maternal nutrition adversely influences the developmental trajectory of the fetal cardiovascular system. These processes collectively increase the offspring’s susceptibility to cardiometabolic diseases in adulthood (88, 103, 106).

**Figure 2:**
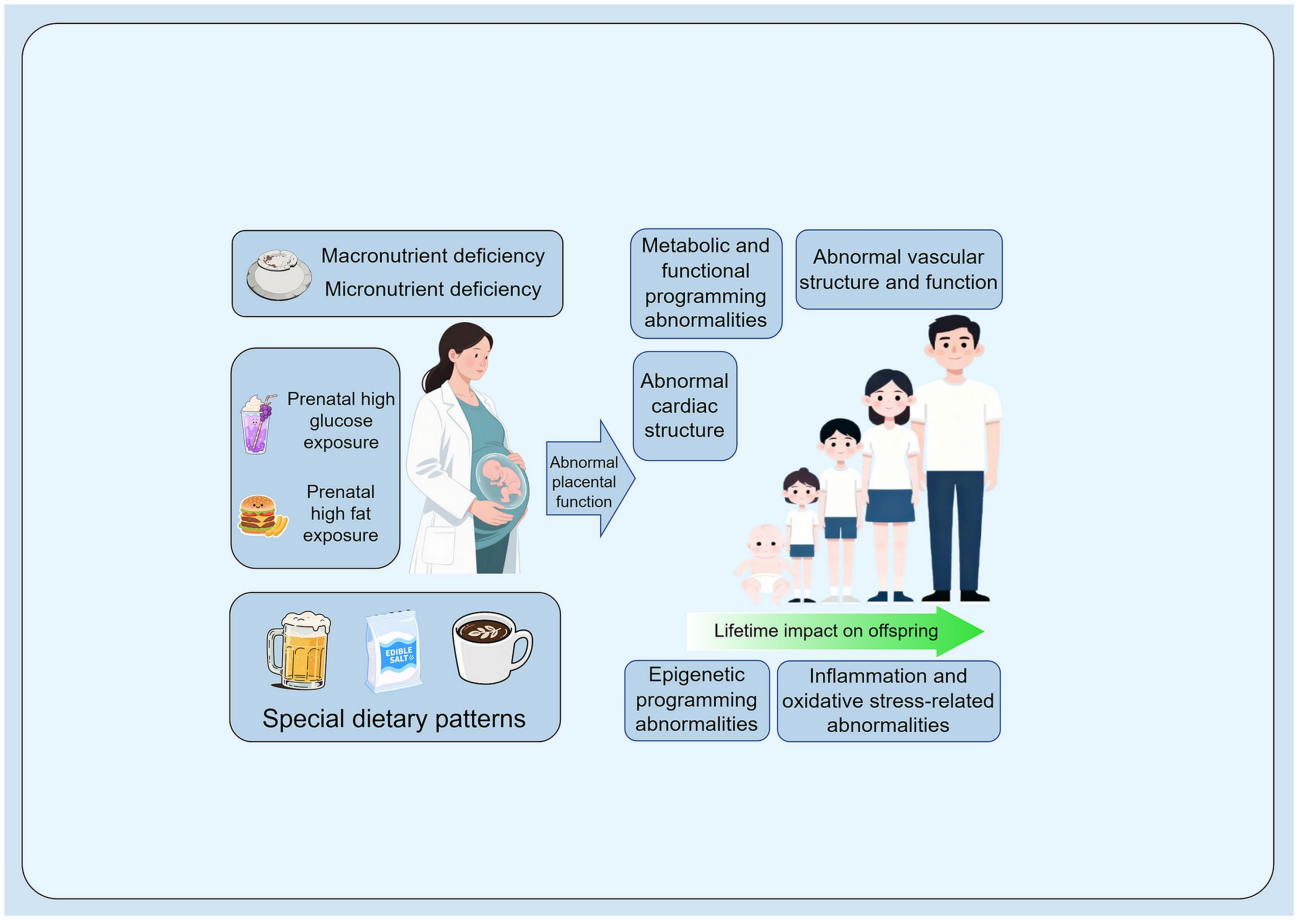
Pathogenic mechanisms linking maternal-specific adverse dietary patterns during pregnancy to fetal-origin cardiovascular disease. Created using Figdraw.com.

This section focuses on examining the potential long-term effects of three typical adverse dietary behaviors during pregnancy—alcohol consumption, high salt intake, and excessive caffeine intake—on offspring cardiovascular health, along with their underlying biological mechanisms. These maternal dietary behaviors disrupt the normal developmental programming of the fetal cardiovascular system, thereby creating long-term health risks for the offspring. Their common pathways of action primarily involve inducing oxidative stress, promoting inflammatory responses, triggering epigenetic modifications, and causing placental dysfunction. Through these mechanisms, fetal cardiovascular development is disrupted, potentially leading to adaptive changes in structure and function. This, in turn, elevates the risk of developing cardiovascular diseases such as hypertension and atherosclerosis in adulthood (Figure 2).

## 6 Conclusion and outlook

**Figure 3:**
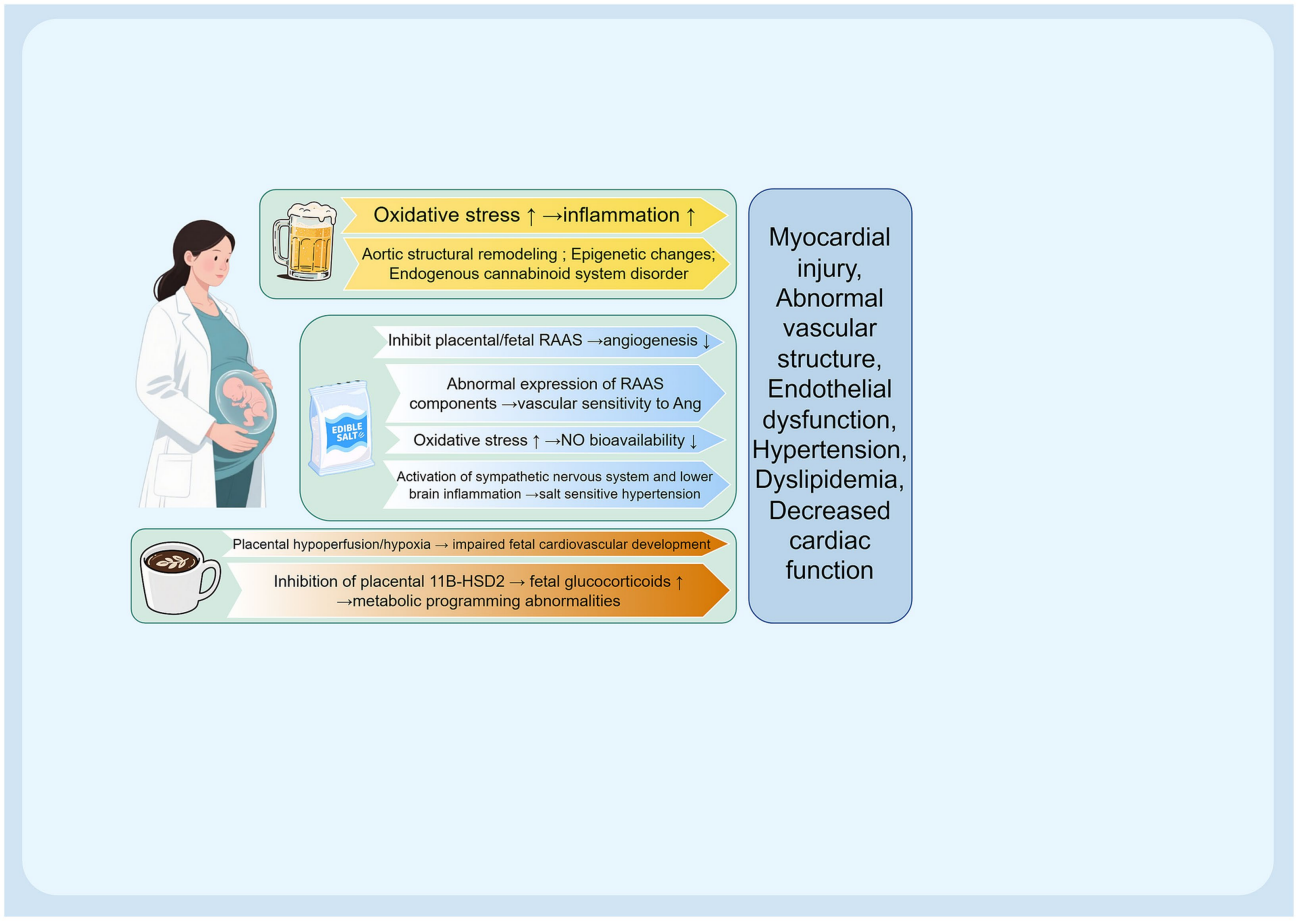
The outline of this review. Created using Figdraw.com.

This review synthesizes evidence that maternal nutritional imbalances during pregnancy—encompassing overnutrition, macro- and micronutrient deficiencies, and specific dietary behaviors—significantly shape the developmental origins of offspring cardiovascular disease (CVD) (Figure 3).

Accumulating evidence identifies “developmental programming” as the core mechanism, wherein suboptimal intrauterine nutrition induces persistent alterations in fetal cardiovascular development (5, 7). Across the diverse exposures discussed, several interconnected mechanistic pathways—including oxidative stress, inflammatory activation, and epigenetic modifications—recur as a common pathogenic network (31, 53, 87), disrupting placental function and fetal programming (34, 95). Beyond these shared mechanisms, specific nutritional insults exhibit distinct emphases: overnutrition is particularly characterized by profound metabolic disturbances and mitochondrial dysfunction (10, 24, 35); macronutrient deficiency drives structural compromises and severe placental insufficiency (48, 51, 52); micronutrient imbalances distinctly disrupt one-carbon metabolism and antioxidant defenses (16, 69, 70); and exposures to alcohol, high salt, and caffeine exert direct teratogenic effects, program neuroendocrine dysregulation, or induce specific placental dysfunction (87, 99, 108).

Maternal nutrition during gestation is thus a key determinant of offspring cardiovascular risk (112). Imbalances during this sensitive period program elevated disease susceptibility later in life. These insights not only deepen our understanding of the developmental origins of CVD but also underscore pregnancy as a strategic window for early intervention (8, 13). Given the growing prevalence of suboptimal prenatal nutrition and its long-term health implications, there is an urgent need to integrate evidence-based nutritional guidance into maternal health programs and chronic disease prevention initiatives. Promoting optimal prenatal nutrition represents a powerful approach to mitigating the global burden of CVD. Future research should aim to elucidate complex nutrient–environment interactions, refine targeted interventions, evaluate long-term outcomes, and support the translation of scientific evidence into public health practice.

## References

[1] World Health Organization. World health statistics 2021: monitoring health for the SDGs, sustainable development goals. (2021). Available online at: https://www.who.int/publications/i/item/9789240027053 (Accessed September 26, 2025).

[2] Sulyok E Farkas B Bodis J. Pathomechanisms of prenatally programmed adult diseases. Antioxidants (Basel). (2023) 12:1354. doi: 10.3390/antiox12071354 37507894 PMC10376205

[3] Khan SS Brewer LC Canobbio MM Cipolla MJ Grobman WA Lewey J . Optimizing prepregnancy cardiovascular health to improve outcomes in pregnant and postpartum individuals and offspring: a scientific statement from the American Heart Association. Circulation. (2023) 147:e76–91. doi: 10.1161/CIR.0000000000001124, 36780391 PMC10080475

[4] Gaillard R Jaddoe VWV. Maternal cardiovascular disorders before and during pregnancy and offspring cardiovascular risk across the life course. Nat Rev Cardiol. (2023) 20:617–30. doi: 10.1038/s41569-023-00869-z, 37169830

[5] Briana DD Malamitsi-Puchner A. Developmental origins of adult health and disease: the metabolic role of BDNF from early life to adulthood. Metabolism. (2018) 81:45–51. doi: 10.1016/j.metabol.2017.11.019, 29217485

[6] Fleming TP Watkins AJ Velazquez MA Mathers JC Prentice AM Stephenson J . Origins of lifetime health around the time of conception: causes and consequences. Lancet. (2018) 391:1842–52. doi: 10.1016/S0140-6736(18)30312-X, 29673874 PMC5975952

[7] Barker DJ. Fetal origins of coronary heart disease. BMJ. (1995) 311:171–4. 7613432 10.1136/bmj.311.6998.171 PMC2550226

[8] Vickers MH. Utility of preclinical models of altered maternal nutrition to support the developmental origins of health and disease hypothesis. Clin Sci Lond. (2022) 136:711–4. doi: 10.1042/CS20211175, 35575180 PMC9112759

[9] Hoffman DJ Powell TL Barrett ES Hardy DB. Developmental origins of metabolic diseases. Physiol Rev. (2021) 101:739–95. doi: 10.1152/physrev.00002.2020, 33270534 PMC8526339

[10] Lippert RN Hess S Klemm P Burgeno LM Jahans-Price T Walton ME . Maternal high-fat diet during lactation reprograms the dopaminergic circuitry in mice. J Clin Invest. (2020) 130:3761–76. doi: 10.1172/JCI134412, 32510473 PMC7324207

[11] Rees WD. Interactions between nutrients in the maternal diet and the implications for the long-term health of the offspring. Proc Nutr Soc. (2019) 78:88–96. doi: 10.1017/S0029665118002537, 30378511

[12] McGee M Bainbridge S Fontaine-Bisson B. A crucial role for maternal dietary methyl donor intake in epigenetic programming and fetal growth outcomes. Nutr Rev. (2018) 76:469–78. doi: 10.1093/nutrit/nuy006, 29529267

[13] Gawlińska K Gawliński D Filip M Przegaliński E. Relationship of maternal high-fat diet during pregnancy and lactation to offspring health. Nutr Rev. (2021) 79:709–25. doi: 10.1093/nutrit/nuaa020, 32447401

[14] Loche E Blackmore HL Carpenter AA Beeson JH Pinnock A Ashmore TJ . Maternal diet-induced obesity programmes cardiac dysfunction in male mice independently of post-weaning diet. Cardiovasc Res. (2018) 114:1372–84. doi: 10.1093/cvr/cvy082, 29635288 PMC6054211

[15] Noble RMN Soni S Liu SN Rachid JJ Mast HE Wiedemeyer A . Maternal ketone supplementation throughout gestation improves neonatal cardiac dysfunction caused by perinatal iron deficiency. Clin Sci Lond. (2024) 138:1249–64. doi: 10.1042/CS20241386, 39288030

[16] Mendes Garrido Abregú F Caniffi C Arranz CT Tomat AL. Impact of zinc deficiency during prenatal and/or postnatal life on cardiovascular and metabolic diseases: experimental and clinical evidence. Adv Nutr. (2022) 13:833–45. doi: 10.1093/advances/nmac012, 35167660 PMC9156367

[17] Chen LW Fitzgerald R Murrin CM Mehegan J Kelleher CC Phillips CM . Associations of maternal caffeine intake with birth outcomes: results from the lifeways cross generation cohort study. Am J Clin Nutr. (2018) 108:1301–8. doi: 10.1093/ajcn/nqy219, 30339199

[18] Polinski KJ Purdue-Smithe A Robinson SL Zhao SK Schliep KC Silver RM . Maternal caffeine intake and DNA methylation in newborn cord blood. Am J Clin Nutr. (2022) 115:482–91. doi: 10.1093/ajcn/nqab348, 34669932 PMC8827095

[19] Fujita T. Recent advances in hypertension: epigenetic mechanism involved in development of salt-sensitive hypertension. Hypertension. (2023) 80:711–8. doi: 10.1161/HYPERTENSIONAHA.122.20588, 36583382

[20] Birukov A Andersen LB Herse F Rakova N Kitlen G Kyhl HB . Aldosterone, salt, and potassium intakes as predictors of pregnancy outcome, including preeclampsia. Hypertension. (2019) 74:391–8. doi: 10.1161/hypertensionaha.119.12924, 31177907

[21] Naik VD Lee J Wu G Washburn S Ramadoss J. Effects of nutrition and gestational alcohol consumption on fetal growth and development. Nutr Rev. (2022) 80:1568–79. doi: 10.1093/nutrit/nuab119, 35092295 PMC9086808

[22] Hossin MZ Kazamia K Faxén J Rudolph A Johansson K Sandström A . Pre-existing maternal cardiovascular disease and the risk of offspring cardiovascular disease from infancy to early adulthood. Eur Heart J. (2024) 45:4111–23. doi: 10.1093/eurheartj/ehae547, 39228375 PMC11458151

[23] Cochrane ALK Murphy MP Ozanne SE Giussani DA. Pregnancy in obese women and mechanisms of increased cardiovascular risk in offspring. Eur Heart J. (2024) 45:5127–45. doi: 10.1093/eurheartj/ehae671, 39508438 PMC11663486

[24] Grzęda E Matuszewska J Ziarniak K Gertig-Kolasa A Krzyśko-Pieczka I Skowrońska B . Animal foetal models of obesity and diabetes - from laboratory to clinical settings. Front Endocrinol (Lausanne). (2022) 13:785674. doi: 10.3389/fendo.2022.785674, 35197931 PMC8858803

[25] Razaz N Villamor E Muraca GM Bonamy AKE Cnattingius S. Maternal obesity and risk of cardiovascular diseases in offspring: a population-based cohort and sibling-controlled study. Lancet Diabetes Endocrinol. (2020) 8:572–81. doi: 10.1016/S2213-8587(20)30151-0, 32559473

[26] Eitmann S Mátrai P Németh D Hegyi P Lukács A Bérczi B . Maternal overnutrition elevates offspring’s blood pressure-a systematic review and meta-analysis. Paediatr Perinat Epidemiol. (2022) 36:276–87. doi: 10.1111/ppe.12859, 35041216 PMC9305555

[27] van Elten TM Karsten MDA van Poppel MNM Geelen A Limpens J Roseboom TJ . Diet and physical activity in pregnancy and offspring’s cardiovascular health: a systematic review. J Dev Orig Health Dis. (2019) 10:286–98. doi: 10.1017/S204017441800082X, 30419991

[28] Bodnar LM Kirkpatrick SI Yu YH Kennedy E Parisi SM Naimi AI. Heterogeneity in the association between a dietary pattern high in fat, sugar, and sodium and adverse pregnancy outcomes by maternal characteristics: a United States pregnancy cohort study. Am J Clin Nutr. (2025) 122:1103–10. doi: 10.1016/j.ajcnut.2025.07.005, 40653273 PMC12674067

[29] Musial B Vaughan OR Fernandez-Twinn DS Voshol P Ozanne SE Fowden AL . A western-style obesogenic diet alters maternal metabolic physiology with consequences for fetal nutrient acquisition in mice. J Physiol. (2017) 595:4875–92. doi: 10.1113/JP273684, 28382681 PMC5509867

[30] Nakano H Fajardo VM Nakano A. The role of glucose in physiological and pathological heart formation. Dev Biol. (2021) 475:222–33. doi: 10.1016/j.ydbio.2021.01.020, 33577830 PMC8107118

[31] Sultan S Sabeeh R. Impact of gestational and type 2 diabetes on fetal endothelial cell miRNA expression. J Diabetes Complicat. (2025) 39:109106. doi: 10.1016/j.jdiacomp.2025.109106, 40482554

[32] Kwon H Jung YJ Lee Y Son GH Kim HO Maeng YS . Impaired angiogenic function of fetal endothelial progenitor cells via PCDH10 in gestational diabetes mellitus. Int J Mol Sci. (2023) 24:16082. doi: 10.3390/ijms242216082 38003275 PMC10671254

[33] Dela Justina V Gonçalves JS de Freitas RA Fonseca AD Volpato GT Tostes RC . Increased O-linked N-acetylglucosamine modification of NF-ΚB and augmented cytokine production in the placentas from hyperglycemic rats. Inflammation. (2017) 40:1773–81. doi: 10.1007/s10753-017-0620-7, 28688099

[34] Lin XH Gao L Tian S Klausen C Guo MX Gao Q . Maternal high-fat-diet exposure is associated with elevated blood pressure and sustained increased leptin levels through epigenetic memory in offspring. Sci Rep. (2021) 11:316. doi: 10.1038/s41598-020-79604-4, 33431976 PMC7801715

[35] Larsen TD Sabey KH Knutson AJ Gandy TCT Louwagie EJ Lauterboeck L . Diabetic pregnancy and maternal high-fat diet impair mitochondrial dynamism in the developing fetal rat heart by sex-specific mechanisms. Int J Mol Sci. (2019) 20:3090. doi: 10.3390/ijms20123090 31242551 PMC6627740

[36] Ahmed A Delgado-Olguin P. Embryonic programming of heart disease in response to obesity during pregnancy. Biochim Biophys Acta Mol basis Dis. (2020) 1866:165402. doi: 10.1016/j.bbadis.2019.01.028, 30759362

[37] Chattergoon NN Bose K Louey S Jonker SS. Lipid exposure leads to metabolic dysfunction in fetal sheep cardiomyocytes. Physiol Rep. (2025) 13:e70386. doi: 10.14814/phy2.70386, 40420618 PMC12106950

[38] Kappen C Kruger C Jones S Herion NJ Salbaum JM. Maternal diet modulates placental nutrient transporter gene expression in a mouse model of diabetic pregnancy. PLoS One. (2019) 14:e0224754. doi: 10.1371/journal.pone.0224754, 31774824 PMC6881028

[39] Yu S Wen Y Li J Zhang H Liu Y. Prenatal lipopolysaccharide exposure promotes dyslipidemia in the male offspring rats. Front Physiol. (2018) 9:542. doi: 10.3389/fphys.2018.00542, 29867579 PMC5964359

[40] Reginatto MW Fontes KN Monteiro VRS Silva NL Andrade CBV Gomes HR . Effect of sublethal prenatal endotoxaemia on murine placental transport systems and lipid homeostasis. Front Microbiol. (2021) 12:706499. doi: 10.3389/fmicb.2021.706499, 34394055 PMC8363225

[41] Alsiraj Y Huang H Shoemaker R Schanbacher B Murphy M Giannone P . Maternal nutritional programming: sex-specific cardiovascular and immune outcomes following perinatal high-fat diet exposure. Nutrients. (2025) 17:1464. doi: 10.3390/nu17091464 40362773 PMC12073119

[42] Duan Y Sun F Li Y Yang S. High glucose and high lipid induced mitochondrial dysfunction in JEG-3 cells through oxidative stress. Open Life Sci. (2023) 18:20220561. doi: 10.1515/biol-2022-0561, 36816801 PMC9922060

[43] Louwagie EJ Larsen TD Wachal AL Baack ML. Placental lipid processing in response to a maternal high-fat diet and diabetes in rats. Pediatr Res. (2018) 83:712–22. doi: 10.1038/pr.2017.288, 29166372 PMC5902636

[44] Contreras-Duarte S Carvajal L Fuenzalida B Cantin C Sobrevia L Leiva A. Maternal dyslipidaemia in pregnancy with gestational diabetes mellitus: possible impact on foetoplacental vascular function and lipoproteins in the neonatal circulation. Curr Vasc Pharmacol. (2019) 17:52–71. doi: 10.2174/1570161115666171116154247, 29149816

[45] Ji B Deng F Zhou B Zhao C Lei J Xu T . Maternal high glucose and fat diet exposure impaired vascular constriction via miR-325-3P/SHIP2/NOX2 pathway axis in offspring vessels. Cell Mol Life Sci. (2024) 82:12. doi: 10.1007/s00018-024-05549-w, 39719480 PMC11668719

[46] Pan HT Xiong YM Zhu HD Shi XL Yu B Ding HG . Proteomics and bioinformatics analysis of cardiovascular related proteins in offspring exposed to gestational diabetes mellitus. Front Cardiovasc Med. (2022) 9:1021112. doi: 10.3389/fcvm.2022.1021112 36277748 PMC9582427

[47] Ricci TA Boonpattrawong N Laher I Devlin AM. Maternal nutrition and effects on offspring vascular function. Pflugers Arch. (2023) 475:877–87. doi: 10.1007/s00424-023-02807-x, 37041303

[48] Van Gronigen Case G Storey KM Parmeley LE Schulz LC. Effects of maternal nutrient restriction during the periconceptional period on placental development in the mouse. PLoS One. (2021) 16:e0244971. doi: 10.1371/journal.pone.0244971, 33444393 PMC7808591

[49] Rock CR White TA Piscopo BR Sutherland AE Miller SL Camm EJ . Cardiovascular and cerebrovascular implications of growth restriction: mechanisms and potential treatments. Int J Mol Sci. (2021) 22:7555. doi: 10.3390/ijms22147555 34299174 PMC8303639

[50] Pereira SP Tavares LC Duarte AI Baldeiras I Cunha-Oliveira T Martins JD . Sex-dependent vulnerability of fetal nonhuman primate cardiac mitochondria to moderate maternal nutrient reduction. Clin Sci (Lond). (2021) 135:1103–26. doi: 10.1042/CS20201339 33899910 PMC8456369

[51] Folguieri MS Franco ATB Vieira AS Gontijo JAR Boer PA. Transcriptome and morphological analysis on the heart in gestational protein-restricted aging male rat offspring. Front Cell Dev Biol. (2022) 10:892322. doi: 10.3389/fcell.2022.892322, 36353510 PMC9638007

[52] Zouridis A Manousopoulou A Potiris A Sarli PM Aravantinos L Pervanidou P . Impact of maternal food restriction on heart proteome in appropriately grown and growth-restricted Wistar-rat offspring. Nutrients. (2021) 13:466. doi: 10.3390/nu13020466 33573223 PMC7912475

[53] de Sousa SM Braz GRF Freitas C d M de Santana DF Sellitti DF Fernandes MP . Oxidative injuries induced by maternal low-protein diet in female brainstem. Nutr Neurosci. (2018) 21:580–8. doi: 10.1080/1028415X.2017.1325974, 28494696

[54] Santana DF Ferreira DS Braz GRF Sousa SMS Silva TL d A Gomes DA . Maternal protein restriction in two successive generations impairs mitochondrial electron coupling in the progeny’s brainstem of Wistar rats from both sexes. Front Neurosci. (2019) 13:203. doi: 10.3389/fnins.2019.00203 30930735 PMC6427765

[55] Makayes Y Resnick E Hinden L Aizenshtein E Shlomi T Kopan R . Increasing mTORC1 pathway activity or methionine supplementation during pregnancy reverses the negative effect of maternal malnutrition on the developing kidney. J Am Soc Nephrol. (2021) 32:1898–912. doi: 10.1681/ASN.2020091321, 33958489 PMC8455268

[56] Mariano VS Boer PA Gontijo JAR. Fetal undernutrition programming, sympathetic nerve activity, and arterial hypertension development. Front Physiol. (2021) 12:704819. doi: 10.3389/fphys.2021.704819, 34867434 PMC8635863

[57] Kawarazaki W Fujita T. Kidney and epigenetic mechanisms of salt-sensitive hypertension. Nat Rev Nephrol. (2021) 17:350–63. doi: 10.1038/s41581-021-00399-2, 33627838

[58] Assalin HB Gontijo JAR Boer PA. miRNAs, target genes expression and morphological analysis on the heart in gestational protein-restricted offspring. PLoS One. (2019) 14:e0210454. doi: 10.1371/journal.pone.0210454, 31034522 PMC6507319

[59] Gallagher LT Bardill J Sucharov CC Wright CJ Karimpour-Fard A Zarate M . Dysregulation of miRNA-mRNA expression in fetal growth restriction in a caloric restricted mouse model. Sci Rep. (2024) 14:5579. doi: 10.1038/s41598-024-56155-6, 38448721 PMC10918062

[60] Masoumy EP Sawyer AA Sharma S Patel JA Gordon PMK Regnault TRH . The lifelong impact of fetal growth restriction on cardiac development. Pediatr Res. (2018) 84:537–44. doi: 10.1038/s41390-018-0069-x, 29967522 PMC6265071

[61] Dimasi CG Darby JRT Cho SKS Saini BS Holman SL Meakin AS . Reduced in utero substrate supply decreases mitochondrial abundance and alters the expression of metabolic signalling molecules in the fetal sheep heart. J Physiol. (2024) 602:5901–22. doi: 10.1113/JP285572, 37996982

[62] Turan S Aberdeen GW Thompson LP. Chronic hypoxia alters maternal uterine and fetal hemodynamics in the full-term pregnant guinea pig. Am J Physiol Regul Integr Comp Physiol. (2017) 313:R330–9. doi: 10.1152/ajpregu.00056.2017, 28679680 PMC5668613

[63] Frapin M Guignard S Meistermann D Grit I Moullé VS Paillé V . Maternal protein restriction in rats alters the expression of genes involved in mitochondrial metabolism and epitranscriptomics in fetal hypothalamus. Nutrients. (2020) 12:1464. doi: 10.3390/nu12051464 32438566 PMC7284977

[64] Masiero BC Calsa B Oliveira CA de Moretti Andra TA Esquisatto MAM Catisti R. Morphofunctional and immunological cardiac evaluation of protein restriction on rat offspring. Ann Anat. (2022) 241:151889. doi: 10.1016/j.aanat.2022.151889 35066148

[65] Miles TK Allensworth-James ML Odle AK Silva Moreira AR Haney AC LaGasse AN . Maternal undernutrition results in transcript changes in male offspring that may promote resistance to high fat diet induced weight gain. Front Endocrinol (Lausanne). (2023) 14:1332959. doi: 10.3389/fendo.2023.1332959, 38720938 PMC11077627

[66] Wang H Mueller NT Li J Sun N Huo Y Ren F . Association of maternal plasma folate and cardiometabolic risk factors in pregnancy with elevated blood pressure of offspring in childhood. Am J Hypertens. (2017) 30:532–40. doi: 10.1093/ajh/hpx003, 28338750 PMC5861539

[67] Sangüesa J Márquez S Montazeri P Fochs S Pey N Anguita-Ruiz A . Role of maternal vitamin D3 levels in shaping adolescent vascular health: evidence from a Spanish population-based birth cohort. J Am Heart Assoc. (2025) 14:e035273. doi: 10.1161/JAHA.124.035273, 40008531 PMC12748081

[68] Lindberg J Norman M Westrup B Domellöf M Berglund SK. Lower systolic blood pressure at age 7 y in low-birth-weight children who received iron supplements in infancy: results from a randomized controlled trial. Am J Clin Nutr. (2017) 106:475–80. doi: 10.3945/ajcn.116.150482, 28659293

[69] Morales E Prieto-Sánchez MT Mendiola J Cutillas-Tolín A Adoamnei E Valera-Gran D . Maternal non-compliance with recommended folic acid supplement use alters global DNA methylation in cord blood of newborns: a cohort study. Clin Nutr. (2024) 43:1191–8. doi: 10.1016/j.clnu.2024.04.007, 38631086

[70] Qu Y Liu X Lin S Bloom MS Wang X Li X . Maternal serum folate during pregnancy and congenital heart disease in offspring. JAMA Netw Open. (2024) 7:e2438747. doi: 10.1001/jamanetworkopen.2024.38747, 39388179 PMC11581582

[71] Kanasaki K Kumagai A. The impact of micronutrient deficiency on pregnancy complications and development origin of health and disease. J Obstet Gynaecol. (2021) 47:1965–72. doi: 10.1111/jog.14770, 33783077

[72] Monasso GS Felix JF Heil SG de Rijke YB Gaillard R Jaddoe VWV. Vitamin B12, folate and homocysteine concentrations during pregnancy and early signs of atherosclerosis at school-age. Clin Nutr. (2021) 40:5133–40. doi: 10.1016/j.clnu.2021.08.001, 34461587 PMC7613758

[73] Huang L Gao W He X Yuan T Zhang H Zhang X . Maternal zinc alleviates tert-butyl hydroperoxide-induced mitochondrial oxidative stress on embryonic development involving the activation of Nrf2/PGC-1α pathway. J Anim Sci Biotechnol. (2023) 14:45. doi: 10.1186/s40104-023-00852-1, 37041604 PMC10091542

[74] Manik KA Joice PPS Jagadal IA T K J Samundeeswari V Madompoyil B . The role of Lp-PLA2 as a mediator between serum magnesium and zinc levels and cardiovascular risk in patients with metabolic syndrome. Cureus. (2024) 16:e72107. doi: 10.7759/cureus.72107 39574996 PMC11580104

[75] Kalisch-Smith JI Ved N Szumska D Munro J Troup M Harris SE . Maternal iron deficiency perturbs embryonic cardiovascular development in mice. Nat Commun. (2021) 12:3447. doi: 10.1038/s41467-021-23660-5, 34103494 PMC8187484

[76] Naowar M Dickton D Francis J. Cardiometabolic risk factors associated with magnesium and vitamin D nutrients during pregnancy-a narrative review. Nutrients. (2024) 16:2630. doi: 10.3390/nu16162630 39203767 PMC11357465

[77] Hu R Huang Y Jiang X Xu Y Zheng Z Shi Y . Maternal dietary copper deficiency induces cardiomyopathy and liver injury in mice by activating autophagy. Nutr Res. (2024) 126:1–10. doi: 10.1016/j.nutres.2024.02.010, 38555686

[78] Monangi NK Xu H Fan YM Khanam R Khan W Deb S . Association of maternal prenatal copper concentration with gestational duration and preterm birth: a multicountry meta-analysis. Am J Clin Nutr. (2024) 119:221–31. doi: 10.1016/j.ajcnut.2023.10.011, 37890672 PMC10808817

[79] Popova S Charness ME Burd L Crawford A Hoyme HE Mukherjee RAS . Fetal alcohol spectrum disorders. Nat Rev Dis Primers. (2023) 9:11. doi: 10.1038/s41572-023-00420-x, 36823161

[80] Jin F Qiao C. Association of maternal caffeine intake during pregnancy with low birth weight, childhood overweight, and obesity: a meta-analysis of cohort studies. Int J Obes. (2021) 45:279–87. doi: 10.1038/s41366-020-0617-4, 32518355

[81] Inoue M Tsuchihashi T Hasuo Y Ogawa M Tominaga M Arakawa K . Salt intake, home blood pressure, and perinatal outcome in pregnant women. Circ J. (2016) 80:2165–72. doi: 10.1253/circj.CJ-16-0405, 27568849

[82] Qian J Chen Q Ward SM Duan E Zhang Y. Impacts of caffeine during pregnancy. Trends Endocrinol Metab. (2020) 31:218–27. doi: 10.1016/j.tem.2019.11.004, 31818639 PMC7035149

[83] Chen LW Murrin CM Mehegan J Kelleher CC Phillips CM. Maternal, but not paternal or grandparental, caffeine intake is associated with childhood obesity and adiposity: the lifeways cross-generation cohort study. Am J Clin Nutr. (2019) 109:1648–55. doi: 10.1093/ajcn/nqz019, 31136661 PMC7484488

[84] Tobiasz AM Duncan JR Bursac Z Sullivan RD Tate DL Dopico AM . The effect of prenatal alcohol exposure on fetal growth and cardiovascular parameters in a baboon model of pregnancy. Reprod Sci. (2018) 25:1116–23. doi: 10.1177/1933719117734317, 28982294 PMC6346348

[85] Dejong K Olyaei A Lo JO. Alcohol use in pregnancy. Clin Obstet Gynecol. (2019) 62:142–55. doi: 10.1097/GRF.0000000000000414, 30575614 PMC7061927

[86] Steane SE Fielding AM Kent NL Andersen I Browne DJ Tejo EN . Maternal choline supplementation in a rat model of periconceptional alcohol exposure: impacts on the fetus and placenta. Alcohol Clin Exp Res. (2021) 45:2130–46. doi: 10.1111/acer.14685, 34342027

[87] Andreu-Fernández V Serra-Delgado M Almeida-Toledano L García-Meseguer À Vieiros M Ramos-Triguero A . Effect of postnatal epigallocatechin-gallate treatment on cardiac function in mice prenatally exposed to alcohol. Antioxidants (Basel). (2023) 12:1067. doi: 10.3390/antiox12051067 37237934 PMC10215419

[88] Shirpoor A Gaderi R Naderi R. Ethanol exposure in prenatal and early postnatal induced cardiac injury in rats: involvement of oxidative stress, Hsp70, ERK 1/2, JNK, and apoptosis in a 3-month follow-up study. Cell Stress Chaperones. (2019) 24:917–26. doi: 10.1007/s12192-019-01015-w, 31410726 PMC6717233

[89] Soraya H Sheikholeslami S Shirpoor A Nezami Majd F Naderi R Rasmi Y. Influence of maternal ethanol exposure on systemic hemodynamic variables and histopathological changes in the aorta wall of male rat offspring: a three-month follow-up. Iran J Med Sci. (2022) 47:468–76. doi: 10.30476/IJMS.2021.91047.2205, 36117576 PMC9445872

[90] Atum ALB de Matos LP de Jesus BC Nasuk GR da Silva GA Gomes CP . Impact of prenatal alcohol exposure on the development and myocardium of adult mice: morphometric changes, transcriptional modulation of genes related to cardiac dysfunction, and antioxidant cardioprotection. Antioxidants (Basel). (2023) 12:256. doi: 10.3390/antiox12020256 36829814 PMC9952294

[91] Helfrich KK Saini N Kwan STC Rivera OC Mooney SM Smith SM. Fetal anemia and elevated hepcidin in a mouse model of fetal alcohol spectrum disorder. Pediatr Res. (2023) 94:503–11. doi: 10.1038/s41390-023-02469-6, 36702950 PMC11878275

[92] Huebner SM Helfrich KK Saini N Blohowiak SE Cheng AA Kling PJ . Dietary iron fortification normalizes fetal hematology, hepcidin, and iron distribution in a rat model of prenatal alcohol exposure. Alcohol Clin Exp Res. (2018) 42:1022–33. doi: 10.1111/acer.13754, 29672865 PMC6317737

[93] Legault LM Dupas T Breton-Larrivée M Filion-Bienvenue F Lemieux A Langford-Avelar A . Sex-specific DNA methylation and gene expression changes in mouse placentas after early preimplantation alcohol exposure. Environ Int. (2024) 192:109014. doi: 10.1016/j.envint.2024.109014, 39321537

[94] Seleverstov O Tobiasz A Jackson JS Sullivan R Ma D Sullivan JP . Maternal alcohol exposure during mid-pregnancy dilates fetal cerebral arteries via endocannabinoid receptors. Alcohol. (2017) 61:51–61. doi: 10.1016/j.alcohol.2017.01.014, 28554529 PMC5517095

[95] Gutherz OR Deyssenroth M Li Q Hao K Jacobson JL Chen J . Potential roles of imprinted genes in the teratogenic effects of alcohol on the placenta, somatic growth, and the developing brain. Exp Neurol. (2022) 347:113919. doi: 10.1016/j.expneurol.2021.113919, 34752786

[96] Vulin M Drenjančević I Muller A Mihaljević Z Kolobarić N Šušnjara P . Placenta may exert fetal protection against maternal high salt diet intake via renin-angiotensin-aldosterone system. Placenta. (2024) 158:136–44. doi: 10.1016/j.placenta.2024.10.003, 39427563

[97] Barakat N Olabi A Nasreddine L Ismaeel H Kharroubi S Jaoude LA . Determination of salt contents of bread types and estimation of salt intake from bread in Lebanon. PLoS One. (2025) 20:e0325857. doi: 10.1371/journal.pone.0325857, 40504794 PMC12161562

[98] Bhattarai S Bista B Yadav BK Gynawali P Poudyal A Jha AK . Estimation of mean population salt intakes using spot urine samples and associations with body mass index, hypertension, raised blood sugar and hypercholesterolemia: findings from STEPS survey 2019, Nepal. PLoS One. (2022) 17:e0266662. doi: 10.1371/journal.pone.0266662, 35413065 PMC9004746

[99] Li W Lv J Wu J Zhou X Jiang L Zhu X . Maternal high-salt diet altered PKC/MLC20 pathway and increased ANG II receptor-mediated vasoconstriction in adult male rat offspring. Mol Nutr Food Res. (2016) 60:1684–94. doi: 10.1002/mnfr.201500998, 26991838

[100] Seravalli P de Oliveira IB Zago BC de Castro I Veras MM Alves-Rodrigues EN . High and low salt intake during pregnancy: impact on cardiac and renal structure in newborns. PLoS One. (2016) 11:e0161598. doi: 10.1371/journal.pone.0161598, 27560182 PMC4999234

[101] Liu Y Qi L Wu J Xu T Yang C Chen X . Prenatal high-salt diet impaired vasodilatation with reprogrammed renin-angiotensin system in offspring rats. J Hypertens. (2018) 36:2369–79. doi: 10.1097/HJH.0000000000001865, 30382958

[102] Vulin M Muller A Drenjančević I Šušnjara P Mihaljević Z Stupin A. High dietary salt intake attenuates nitric oxide mediated endothelium-dependent vasodilation and increases oxidative stress in pregnancy. J Hypertens. (2024) 42:672–84. doi: 10.1097/HJH.0000000000003645, 38230612

[103] Su Q Yu XJ Yang Q Wang XM Xia WJ Li HB . Inhibition of maternal c-Src ameliorates the male offspring hypertension by suppressing inflammation and neurotransmitters in the paraventricular nucleus. Cardiovasc Toxicol. (2021) 21:820–34. doi: 10.1007/s12012-021-09672-z, 34269955

[104] Kukkonen A Hantunen S Voutilainen A Ruusunen A Backman K Kirjavainen PV . Maternal caffeine intake during pregnancy and the risk of delivering a small for gestational age baby: Kuopio birth cohort. Arch Gynecol Obstet. (2024) 310:359–68. doi: 10.1007/s00404-024-07538-7, 38767721 PMC11169027

[105] Kobayashi S Sata F Murata K Saijo Y Araki A Miyashita C . Dose-dependent associations between prenatal caffeine consumption and small for gestational age, preterm birth, and reduced birthweight in the Japan environment and children’s study. Paediatr Perinat Epidemiol. (2019) 33:185–94. doi: 10.1111/ppe.12551, 31020683

[106] Xu D Luo HW Hu W Hu SW Yuan C Wang GH . Intrauterine programming mechanism for hypercholesterolemia in prenatal caffeine-exposed female adult rat offspring. FASEB J. (2018) 32:5563–76. doi: 10.1096/fj.201701557R, 29718709

[107] Hu S Liu K Luo H Xu D Chen L Zhang L . Caffeine programs hepatic SIRT1-related cholesterol synthesis and hypercholesterolemia via A2AR/cAMP/PKA pathway in adult male offspring rats. Toxicology. (2019) 418:11–21. doi: 10.1016/j.tox.2019.02.015, 30825513

[108] Li N Shi R Tang J Zhang W Liu B Chen X . Prenatal caffeine exposure induces down-regulation of the protein kinase a/ryanodine receptor/large-conductance Ca2+−activated K+ pathway in the cerebral arteries of old offspring rats. J Hypertens. (2020) 38:679–91. doi: 10.1097/HJH.0000000000002303, 31834119

[109] Fang X Poulsen RR Rivkees SA Wendler CC. In utero caffeine exposure induces transgenerational effects on the adult heart. Sci Rep. (2016) 6:34106. doi: 10.1038/srep34106 27677355 PMC5039698

[110] Yu P Zhou J Ge C Fang M Zhang Y Wang H. Differential expression of placental 11β-HSD2 induced by high maternal glucocorticoid exposure mediates sex differences in placental and fetal development. Sci Total Environ. (2022) 827:154396.doi: 10.1016/j.scitotenv.2022.154396 35259391

[111] Paula T d MDE Cardoso LC Felicioni F Caldeira-Brant AL Santos TG Castro-Oliveira H . Maternal chronic caffeine intake impairs fertility, placental vascularization and fetal development in mice. Reprod Toxicol. (2023) 121:108471. doi: 10.1016/j.reprotox.2023.108471, 37717671

[112] Jiao P Lu H Hao L Degen AA Cheng J Yin Z . Nutrigenetic and epigenetic mechanisms of maternal nutrition-induced glucolipid metabolism changes in the offspring. Nutr Rev. (2025) 83:728–48. doi: 10.1093/nutrit/nuae048, 38781288

